# Microalgae–material hybrid for enhanced photosynthetic energy conversion: a promising path towards carbon neutrality

**DOI:** 10.1093/nsr/nwad200

**Published:** 2023-07-18

**Authors:** Wei Xiong, Yiyan Peng, Weimin Ma, Xurong Xu, Yueqi Zhao, Jinhui Wu, Ruikang Tang

**Affiliations:** School of Chemistry and Chemical Engineering, Nanchang University, Nanchang 330031, China; School of Chemistry and Chemical Engineering, Nanchang University, Nanchang 330031, China; College of Life Sciences, Shanghai Normal University, Shanghai 200234, China; Department of Chemistry, Zhejiang University, Hangzhou 310058, China; Qiushi Academy for Advanced Studies, Zhejiang University, Hangzhou 310027, China; Department of Orthopaedic Surgery, Sir Run Run Shaw Hospital, School of Medicine, Zhejiang University, Hangzhou 310016, China; State Key Laboratory of Pharmaceutical Biotechnology, Medical School & School of Life Sciences, Nanjing University, Nanjing 210093, China; Department of Chemistry, Zhejiang University, Hangzhou 310058, China; Qiushi Academy for Advanced Studies, Zhejiang University, Hangzhou 310027, China

**Keywords:** microalgae–material hybrid, photosynthesis, cell–material interaction, energy conversion, carbon neutrality

## Abstract

Photosynthetic energy conversion for high-energy chemicals generation is one of the most viable solutions in the quest for sustainable energy towards carbon neutrality. Microalgae are fascinating photosynthetic organisms, which can directly convert solar energy into chemical energy and electrical energy. However, microalgal photosynthetic energy has not yet been applied on a large scale due to the limitation of their own characteristics. Researchers have been inspired to couple microalgae with synthetic materials via biomimetic assembly and the resulting microalgae–material hybrids have become more robust and even perform new functions. In the past decade, great progress has been made in microalgae–material hybrids, such as photosynthetic carbon dioxide fixation, photosynthetic hydrogen production, photoelectrochemical energy conversion and even biochemical energy conversion for biomedical therapy. The microalgae–material hybrid offers opportunities to promote artificially enhanced photosynthesis research and synchronously inspires investigation of biotic–abiotic interface manipulation. This review summarizes current construction methods of microalgae–material hybrids and highlights their implication in energy and health. Moreover, we discuss the current problems and future challenges for microalgae–material hybrids and the outlook for their development and applications. This review will provide inspiration for the rational design of the microalgae-based semi-natural biohybrid and further promote the disciplinary fusion of material science and biological science.

## Teaser

Microalgae-material hybrid offers great prospects in the fields of clean energy, environmental protection, and life health, which contributes to the realization of carbon peaking and carbon neutrality.

## INTRODUCTION

Microalgae, including cyanobacteria and green algae, represent the most important biological systems for producing biomass and high-value products [1]. As the most important primary producer, microalgae are found almost everywhere and have a profound impact on Earth's atmosphere and biosphere [2,3]. Currently, excessive exploitation and utilization of fossil fuels have produced a large amount of carbon dioxide emissions, contributing to increasing global warming. Climate change is a crisis facing mankind, while carbon neutrality is a common interest and responsibility of mankind. The Chinese government has announced to the world a timetable for carbon peaking and carbon neutrality—that is, China aims to peak its carbon dioxide emissions before 2030 and strives to achieve carbon neutrality before 2060. Energy transition is the fundamental guarantee for the realization of carbon-peaking and carbon-neutrality targets. Photosynthesis can convert carbon dioxide and water into biomass and valuable products, which is one of the most important pathways for carbon neutrality in nature. Microalgal photosynthesis could not only absorb the carbon being put out by fossil fuel combustion, but also produce hydrogen that is fully carbon-neutral [4,5]. Further, microalgae are suitable raw materials for photosynthesis-derived fuels like biodiesel, biohydrogen and bioethanol [6] because their cultivation does not need to occupy arable land and can use saltwater and wastewater [7]. Therefore, exploiting microalgae photosynthetic energy is a promising approach to energy transition for carbon peak and neutrality.

Microalgae production technologies, including microalgae selection, breeding and engineering, are playing a more and more important role in biofuel production [8]. However, the photosynthetic conversion efficiency is a major limitation for microalgae biofuels production. Generally, wild-type strains of microalgae can capture 43% of the sunlight energy in the photosynthesis, but only ∼4–8% of the light energy can be converted into chemical energy in the form of biomass (theoretical maximum is 9%) [9]. Therefore, the microalgae production of biofuels cannot meet human energy needs. Genetic engineering and metabolic engineering can be used to improve the microalgal production of biomass and lipids [10] but these methods require complicated operation and high cost with a narrow scope for application.

**Figure 1.**
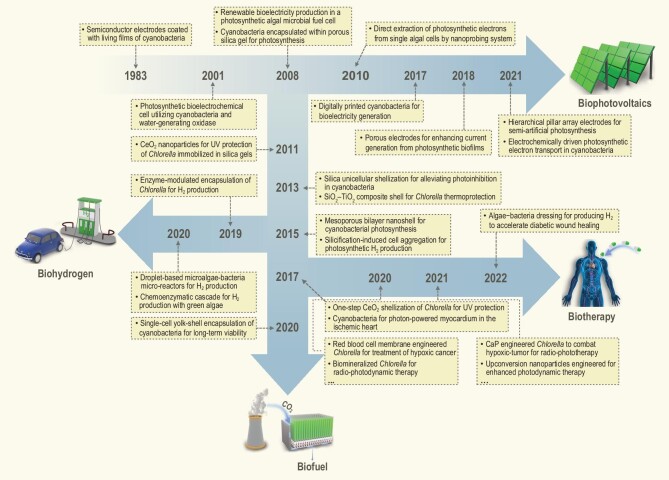
Timeline of the development of a microalgae–material hybrid for enhanced photosynthetic energy conversion.

In nature, organisms can form organic–inorganic composite materials with complex structures and excellent biological properties through biomineralization, such as bones, teeth and shells [11,12]. These biomaterials have a highly ordered hierarchical structure from nanoscale to macroscale, which can provide organisms with functions such as mechanical support, protection, movement and signal sensing [12–15]. For example, diatoms can use silicate in the environment to form a finely structured silica shell on the cell surface, which can provide them with mechanical protection, photonic crystals and pH buffers [16,17]. In addition, there are some bacteria that can use internal magnetite crystals (Fe_3_O_4_ or Fe_3_S_4_) as magnetic sensors [13,18]. Inspired by the natural biomineralization phenomenon, an artificial cell–material hybrid has received increasing interest for green chemistry and engineered living biomaterials [19,20]. In order to exploit the microalgal photosynthetic energy, scientists have been inspired to interface nature's photosynthetic organisms with synthetic materials so as to impart the organisms with new properties. We define the artificially generated biomorph as a microalgae–material hybrid (MMH), which is mainly based upon microscale interactions, such as chemical bonds or non-covalent interactions. In the early research stage, the silica sol–gel method was used for immobilizing cyanobacteria to enhance photosynthetic CO_2_ fixation [21]. In pursuit of new photosynthetic energy conversion, silicification-induced multicellular aggregation was developed for inducing photosynthetic H_2_ production under aerobic conditions [22]. Further, cyanobacteria have been coupled with electrodes for bioelectrochemical energy conversion [23]. These MMHs offer a great incentive to develop semi-natural photosynthetic systems for solar-to-chemical energy conversion. In addition, the MMHs have recently been used in photo-driven biomedical therapy due to their characteristics of photosynthetic H_2_ or O_2_ evolution [24,25]. It follows that the use of MMHs for biological regulation is becoming an emerging field. In this feature article, we review the recent achievements in the material-based modification of microalgae, as well as their application in solar energy utilization, water environment protection and biomedical therapy (Fig. 1). Furthermore, we elucidate the mechanisms of MMH for functional regulation from the perspective of the cell–material interfacial interaction. We also discuss the current problems and outlook for future challenges of MMHs for enhanced photosynthetic energy conversion.

## CONSTRUCTION OF THE MMH

In nature, many organisms have evolved to utilize inorganic materials to achieve optimal function or protect themselves against harsh environments. Among them, diatoms are the typical cell–material hybrids, which can form sophisticated nanostructured silica shells for mechanical protection and photonic response [15]. However, most microalgae cannot spontaneously form cell–material hybrids due to the lack of biomineralization-relevant proteins. In the recent decade, scientists have explored three material approaches to improve microalgae.

### Cell immobilization

The long-term stability of cell activity is crucial for microalgal applications but microalgal cells are usually sensitive to a disturbed environment. Cell immobilization can confer high stability and extend the lifetime for microalgae because the matrix for immobilization provides a stable microenvironment that can protect the cell from harsh external environments, such as temperature fluctuations, pH fluctuations, organic solvents, shear forces and toxins [26–28]. Furthermore, the immobilization can offer easy regulation of the culture environment for cells [26]. In addition, cell immobilization can afford the system a high cell density.

**Figure 2.**
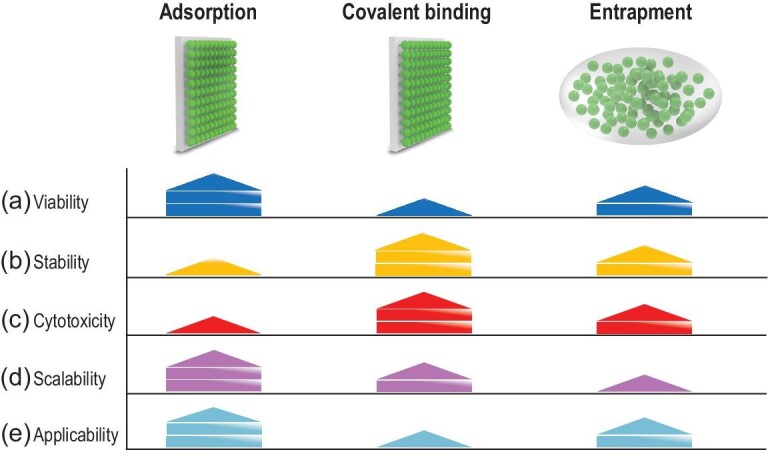
Strengths and limitations of microalgal immobilization methods. Each method features inherent unique functionalities and tradeoffs (a–e) in photosynthetic energy conversion. (a) Viability represents the cell survival state, which can be evaluated by green fluorescence intensity by fluorescein diacetate (FDA)-stained cells under confocal laser scanning microscopy. (b) Stability represents the durability of the microalgae–material interaction, which can be evaluated by the maintenance time of the hybrid structure. (c) Cytotoxicity represents the effect of the microalgae–material interaction on cell viability, which can be evaluated by MTT assay or a fluorescein luminescence method. (d) Scalability indicates the potential for large-scale microalgae immobilization. (e) Applicability indicates the application prospect of the immobilized microalgae in a comprehensive consideration of the above factors. Achieving the optimal outcome relies on the rational matching of material properties and microalgal functions. Furthermore, different levels of microalgae–material hybridization may be necessary, depending on the desired outcome.

Microalgal cells could be immobilized by different material strategies. Over the years, three cell immobilization methods such as adsorption, covalent binding and entrapment have been successfully developed [26] (Fig. 2). Adsorption is a mild and reversible process that depends on the physical binding between the cell surface and the material surface, such as van der Waals interactions, ionic forces and hydrogen bonds. Also, immobilization by adsorption permits the reuse of microalgae cells. However, easy leaching limits the development and practical applications of this method. Unlike adsorption, covalent bonding is an irreversible process that can improve the stability of a cell–material hybrid but may have a negative effect on cell activity (Fig. 2). Covalent binding immobilization is based on the covalent bond between cells and the support matrix in the presence of a binding agent. The binding agents are usually cytotoxic and can cause loss of cell viability (Fig. 2) due to conformational transitions or structural changes of organic molecules on the cell surface [26,29]. Therefore, the covalent binding method is not very suitable for whole-cell immobilization of microalgae. Entrapment is a promising way to preserve biosystems [27,30] that is usually based on the soft synthesis of inorganic materials with biocompatible precursors. The advantages of cell entrapment include an easy synthesis process, tunable porosity and good mechanical strength and stability (Fig. 2). For example, the generated porous structures produced by the sol–gel method can provide entrapped cells with an aqueous environment and physiological stability. However, the disadvantages of cell entrapment are also obvious, such as the limitation of cell proliferation and scale application. Generally, cell entrapment is the most appropriate immobilization method for enhancing the stability of microalgae but functional modification of microalgae by materials is limited.

The selection of the material is key to the immobilization process, as it affects the viability and functionality of microalgae. First of all, the biocompatibility of the material is the primary consideration, which means that the material should provide an inert environment for the immobilized cells to keep the biological activity of the enzymes. Second, the cost is also important for material selection, for it directly affects application prospects. Currently, three types of materials are available for efficient immobilization, including inorganic materials, organic materials and composite materials [24]. Inorganic materials include sol–gel-based materials, carbon materials, and oxide and metallic nanomaterials [21,31,32], while organic materials involve natural materials (chitin, agar, alginate, etc.) and synthetic materials (polyvinyl alcohol, polypropylene ammonium, polyurethane, etc.) [33]. The composite materials represent a combination of organic and inorganic materials, which provides the integration of the two types of performance, implying the superiority of composite materials [26]. According to different properties, polymers, carbons, oxide and metallic nanomaterials, and composite materials are usually used in adsorption and covalent binding [26,33], while sol–gel-based materials are mainly used in cell entrapment [21,26].

### Unicellular shellization

Unicellular shellization is an emerging technique of cell-surface engineering [34,35] that emphasizes the protection of living cells against external stresses *in vivo*. Compared with cell immobilization, unicellular shellization allows cell density control and cell behavior monitoring [34,35]. Inspired by biomineralization, cell-in-shell structures are formed through biomimetic mineralization on the cell surface, which can suppress or retardate cell growth and division, but enhance cell survival under harsh conditions. Such a hybrid method facilitates light penetration and mass transfer from the external environment to the enclosed cells, which is suitable for photosynthetic microorganisms. In nature, most microalgae exist as single cells, so unicellular shellization can offer a new approach to improving microalgae. However, it is a great challenge to confer microalgal cells with artificial shells due to the lack of ‘mineralization sites’.

**Figure 3.**
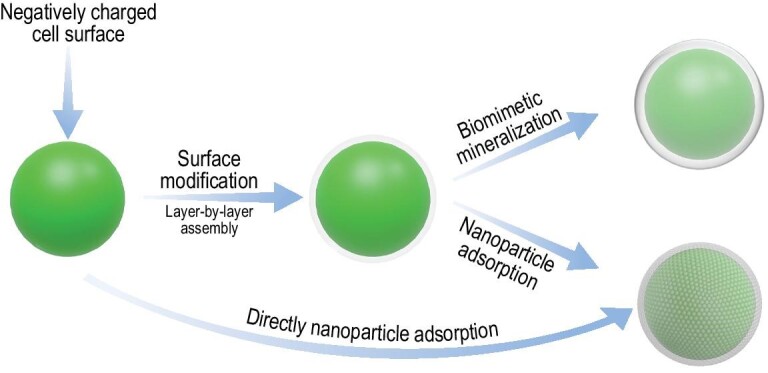
Schematic illustration of microalgal unicellular shellization.

In order to construct artificial shells for microalgae, mineralization-related molecules have to be introduced onto the cell surface. Layer-by-layer (LbL) assembly is an efficient method for cell-surface modification, which can form thin films on the cell surface by depositing alternating layers of oppositely charged or molecular interactive materials [36–38]. The microalgal cell surface is usually negatively charged, so positive polyelectrolytes can be absorbed onto the cell surface to form an electropositive layer (Fig. 3). In the assembled layers, the polyelectrolytes act as ‘branch’ that not only connects the underlying oppositely charged polyelectrolytes, but also increases the functional groups on the cell surface. Inspired by diatoms, an artificial biosilica shell was first introduced onto a cyanobacterial surface with the help of poly(diallyldimethylammonium chloride) (PDADMAC—a cationic polyelectrolyte with positively charged quaternary amines) and poly(styrene sulfonate) (PSS—a biocompatible polyelectrolyte with a negative charge) [39]. PDADMAC was first introduced onto the cyanobacterial surface [39] then PSS was absorbed onto the PDADMAC layer to form an electronegative surface layer. By alternating the two polyelectrolytes with several cycles, a nanofilm with abundant quaternary amines can be formed on the cyanobacterial cell surface and the exposed PDADMAC acted as a catalytic template to induce the silicification on the cell surface. Besides, other functionalized silica shells can also be conferred on a microalgae cell with the help of a modified LbL technique, such as *Chlorella* with a SiO_2_/TiO_2_ shell [40] and cyanobacteria with a mesoporous silica shell [41].

Currently, LbL-assisted silica shellization is one of the most frequently used material technologies to improve microalgae at the single-cell level (Fig. 3). However, excess polyelectrolytes during each polyelectrolyte deposition treatment need to be removed by centrifugation and separation, which are complicated and time-consuming. Moreover, the treatment processes may induce unexpected cell injuries. Therefore, LbL is commonly suitable for robust cells with rigid cell-wall structures such as cyanobacteria and *Chlorella*, but may frequently cause damage to the cell membrane of green algae without cell walls, which are sensitive to chemical and environmental changes. Direct adsorption of nanoparticles is another tactic to improve microalgae for shell formations (Fig. 3), such as cerium dioxide nanoshell for *Chlorella* [42], which is based on electrostatic interaction. The electrostatic interaction mainly depends on the zeta potentials of both the nanoparticle and the cell surface. As the surface of microalgae cells is usually negatively charged, direct adsorption usually needs positively charged nanoparticles. Generally, unicellular shellization is an approach to making microalgae more robust but current methods cannot be applied on a large scale.

### Multicellular aggregation

**Figure 4.**
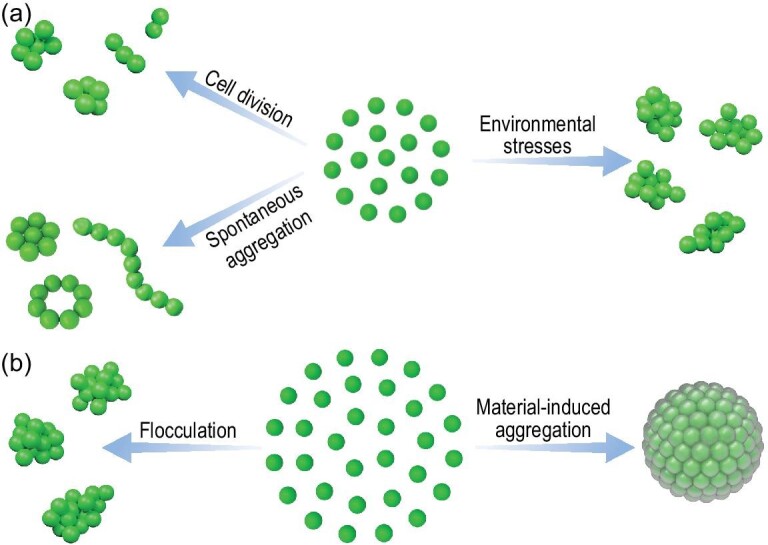
Schematic illustration of microalgal multicellularity and cell aggregation. (a) Common microalgal multicellularity mechanisms in nature. (b) Artificially induced microalgal multicellularity mechanisms.

Multicellularity was a transformative evolution for life on Earth that greatly increased biological complexity through forming new biological structures [43,44]. For example, multicellular organisms have evolved sophisticated functionality via cell cooperation with complementary behaviors [44]. The multicellularity of cyanobacteria evolved nitrogen fixation function [45], while the multicellularity of green algae eventually led to the emergence of plants [46,47]. Therefore, multicellular transition is a promising approach to trigger new functions of microalgae. There are essentially two ways to make a simple multicellular entity out of a single cell: (i) a single cell divides and its offspring stick together; (ii) several solitary cells aggregate to form a colony (Fig. 4a) [44,48]. In nature, some environmental stresses can induce many unicellular microalgal cells to form clusters (Fig. 4a) [49]. For example, predation by a small-mouthed ciliate can result in the generation of cellular clusters of the previously single-celled *Chlorella* [50]. Currently, most approaches to experimental transitions of multicellularity focus on the regulation of ecological conditions that would favor the formation of cellular clusters [51]. These approaches to form cellular clusters are induced by environmental stresses and controlled by genes, but they lack universality, are always time-consuming and are usually based on cell lineages with established multicellular developmental programs or lack of separation following a cell division [52,53]. Hence artificially induced multicellular aggregation is desired for microalgae functionalization.

Artificially induced multicellular aggregation can be achieved by cell flocculation and mineralization-induced cell aggregation. Flocculation is a common method to form larger aggregates from single cells. Approaches for microalgal flocculation range from chemical flocculation to biological flocculation and particle-based flocculation [54]. Chemical flocculation results in toxicity to microalgae, although the use of natural polymeric flocculants may minimize this problem [55]. Biological flocculation using fungi or bacteria holds huge potential in microalgal wastewater treatment because wastewater usually contains a carbon source necessary for the flocculating microorganisms [54]. However, the operation cost needs to be reduced for scalability. Particle-based flocculation using aminoclay-based nanoparticles, magnetic particles or multifunctional particles can overcome some shortcomings of chemical flocculation such as bio-toxicity and difficulty in chemical recycling, but the manufacturing costs of particles could be a major limiting factor for scalable application [54]. All the above approaches of flocculation-induced cell aggregation are based on electrostatic interaction (Fig. 4b) but cannot regulate the structure of cell aggregates. Flocculation has been widely used as a harvesting method for microalgae biomass but the effect of flocculation on the physiological function of microalgae was rarely concerned about. Fortunately, we found that biomimetic silicification can induce cell aggregation of *Chlorella* [22]. The silicification-induced cell aggregation also needs PDADMAC to endow the cell surface with silicification sites following the transformation of the cell-surface potential. Silica can spontaneously cohere PDADMAC-coated *Chlorella* cells together when silicification occurs. Compared with flocculation, silicification-induced cell aggregation can form more compact and stable MMH and may cause more cell damage. Mineralization-induced multicellular aggregation is becoming an emerging method for cell functional transformation, with broad application prospects to explore. Anyway, material-induced multicellular aggregation offers a new pathway to improve microalgae.

## ENHANCEMENT OF PHOTOSYNTHETIC ENERGY CONVERSION BY MMH

### Improving photosynthetic CO_2_ fixation

**Figure 5.**
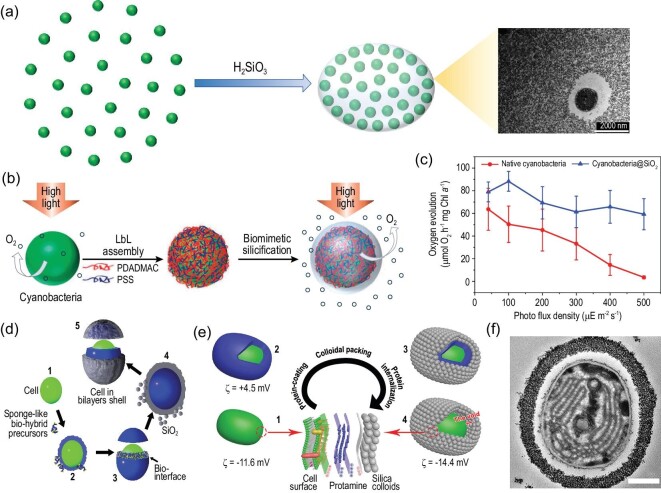
Microalgae–material hybrid for photosynthetic CO_2_ fixation. (a) Schematic illustration and transmission electron microscopy (TEM) micrograph of cyanobacterium *Synechocystis* sp. strain PCC 7002 entrapped within porous silica. Reproduced with permission from [60]. Copyright 2008 Royal Society of Chemistry. (b) Schematic illustration of cyanobacterial silicification for alleviation of photoinhibition. Reproduced with permission from [39]. Copyright 2013 Royal Society of Chemistry. (c) Comparison of photosynthetic production of O_2_ in native cyanobacteria and cyanobacteria@SiO_2_ cells. Reproduced with permission from [39]. Copyright 2013 Royal Society of Chemistry. (d) Schematic illustration of mesoporous bilayer nano shell construction for cyanobacteria. Reproduced with permission from [61]. Copyright 2015 John Wiley & Sons, Inc. (e) Schematic illustration of protamine-assisted silica colloidal packing for cyanobacterial encapsulation [41]. (f) TEM micrograph of a nanosilica-encapsulated cyanobacterium with yolk–shell structure. Scale bar: 500 nm [41].

Microalgal photosynthesis has attracted great attention due to its potential role in renewable energy development [56]. The photosynthetic energy conversion of microalgae could be exploited to reduce CO_2_ emissions, synthesize valuable molecules, decompose toxic chemicals and extract heavy metals [1,57,58]. For example, an estimated 25 Giga tons per year of carbon from CO_2_ can be fixed into biomass by cyanobacteria on a global scale [59]. However, the utilization of microalgal photosynthetic CO_2_ fixation is limited by cell stability and reusability. In order to develop a photosynthetic biosystem suitable for CO_2_ fixation assimilation, Su and his co-workers first entrapped cyanobacterial cells into a porous silica network to enhance the sustainability of the cells (Fig. 5a) [60]. The immobilization step could be achieved via the acidification of aqueous colloidal silica precursors [60]. The immobilization of cyanobacteria created large voids around the cell (Fig. 5a) [60], which could provide space for cell division. Thus, the macrovoids within the mesoporous material would be an ideal microenvironment for cell preservation. The immobilized cyanobacterial cells continued to autofluoresce even after 12 weeks [60], indicating the extended stability of chlorophyll *a* within the immobilized cells and the ability of photosynthesis. Further, the uptake of radio-labeled NaH^14^CO_3_ indicated that the cyanobacterial cells fixed CO_2_ within the silica gel continuously [60]. Cyanobacterial immobilization with silica gel was a pioneering attempt in using microalgae–material hybrids for photosynthetic energy conversion, which opened a window for improving photosynthetic CO_2_ fixation. It should be emphasized that cell immobilization with porous silica gel is a feasible method for cell storage of microalgae due to the cytocompatibility and transparency of silica material, which mainly improves photosynthetic CO_2_ fixation by prolonging cell stability and life. However, the photosynthetic activities and proliferation capacity are inevitably limited compared with the native state.

Currently, unicellular shellization is considered as an efficient way to improve cell activity and function for enhanced ability to adapt to the environment. Inspired by diatoms, we developed a modified LbL method to deposit PDADMAC with the help of PSS [39] (Fig. 5b) and the exposed quaternary amines acted as catalytic sites to induce *in situ* silica shellization (Fig. 5b). The thickness of the silica shell layer was ∼30 nm and the thickness of the shell can be regulated by the number of multilayers [39]. The silica shell can reduce visible light transmission to alleviate high-light stress on the cell [39]. Specifically, the effect of alleviation in silicified cyanobacteria (cyanobacteria@SiO_2_) gradually enhanced with the increase in light intensity, especially under high-light conditions (Fig. 5c). For example, cyanobacteria@SiO_2_ retained ≥50% of the photosynthetic oxygen evolution capacity after exposure to an intense light intensity of 500 μE m^−2^ s^−1^ for 2 h (Fig. 5c). Further, silica-coated cyanobacteria show lower respiratory rates than native cyanobacteria [39]. Therefore, such a silica-based improvement indicates the potential of greatly enhancing the photosynthetic efficiency of cyanobacteria under high-light conditions, which promotes the photosynthetic production of biomass. Unicellular silica shellization can enhance environmental stability without restricting photosynthesis. However, the LbL-based cell shellization requires multiple steps of centrifugation, which are time-consuming and harmful to cell activity. Further, the interface between the coating materials and cells usually has an adverse effect on the cell stability.

In order to enhance both the activity and the stability of the entrapped cells, Su and co-workers constructed a mesoporous silica shell for the cyanobacterial cell using the self-assembled Au@L-cysteine biohybrid layer and silica precursors [61] (Fig. 5d). The worm-like mesoporous silica shell possesses a high surface area with good mass-diffusion ability due to its nanosized channel structure. Hence, the nanoporous structure can allow the transfer of nutrients and chemicals into cells and the bilayer shell can block the excessive light that may cause cell damage [61]. The L-cysteine molecules in the biointerface can buffer the extracellular microenvironment when the acid/base chemical environment is abruptly changed. Cyanobacteria with the mesoporous bilayer nanoshell performed with higher activity and stability than silica-immobilized cyanobacteria. For example, at Day 20, the cells within the mesoporous bilayer shells presented 186% (±17%) of the initial activity and the viabilities of the shell-encapsulated cells were well preserved for ≥50 days under high-light conditions [61]. This work offers a strategy that can construct a multifunctional nanoshell on a cyanobacterial cell surface with high activity and stability. Based on the strategy, Su and co-workers further develop ordered packing of silica nanocolloids for single-cell encapsulation of cyanobacteria [41]. A space between the capsule shell and the cell was created by the internalization of protamine, resulting in a highly ordered porous yolk–shell structure (Fig. 5e and f). The yolk–shell nanostructure leads to a high cell viability of >90% and the rate of photosynthetic oxygen evolution of the yolk–shell-encapsulated cyanobacteria is 3- to 4-fold higher than the cell-contacting shell-encapsulated cyanobacteria under a large range of light intensities (0–500 μE m^−2^ s^−1^) [41]. In addition, the ordered yolk–shell structure can offer the cyanobacterial cell superior protection against harsh external stress, such as resistance against high temperatures. Although the yolk–shell structure was constructed on a cyanobacterial cell, the strategy could potentially be further used in other aspects of biotechnology, such as biosensors, biocatalysis and controlled delivery therapeutics. The studies of Su and co-workers show that the evolution of material technology can enhance the functionalities of microalgae–material hybrids due to the structure and properties of the materials.

Ultraviolet radiation (UV) is also a significant inhibitory condition for microalgae in the sunlight, the biological effects of which mainly involve the formation of reactive oxygen species (ROS) [62]. CeO_2_ nanoparticle-loaded silica hydrogels have been used as UV protective matrices for long-term cell entrapment of *Chlorella vulgaris* [63]. Further, a one-step approach was developed to construct a CeO_2_ nanoshell for *Chlorella* cells by the direct adsorption of CeO_2_ nanoparticles [42], which can efficiently protect the enclosed *Chlorella* cell from UV-induced oxidative stress [42]. In addition, an SiO_2_–TiO_2_ composite shell can also improve photosynthetic activity by relieving heat stress [40].

Currently, MMHs improve photosynthetic carbon fixation mainly by enhancing resistance to environmental stresses. Silica is the most commonly used material due to superior biocompatibility and transparency. However, whether the silica-based cell immobilization technique is applicable for all types of microalgae still needs more cases to verify. It should be noted that silica-based cell immobilization or shellization can enhance the activity and stability of microalgae but it is hard to endow microalgae with functions that they do not have. A microalgae–silica hybrid can extend the living environment of algae from lakes and oceans to land, and has great potential to be applied to desertification control and outer wilds in the future. Although the existing construction methods are not suitable for large-scale applications in pursuit of carbon neutrality, the microalgae–material interface technology may play an important role on some special occasions when cost is not the main consideration.

### Inducing photosynthetic hydrogen production

In nature, photosynthetic microalgae can photolyse water to produce H_2_ and O_2_ using photosynthetic machinery coupled with hydrogenase [64], which is renewable and carbon-neutral. However, this is a transient process because the oxygen generated by the photosystem quickly inactivates hydrogenase [64]. Currently, suppression of O_2_ production and timely removal of O_2_ are the main strategies to generate an anaerobic environment for photosynthetic H_2_ production. Sulfur deprivation is the most commonly used method to suppress O_2_ production of green algae [65] but it would simultaneously suppress photosystem II (PSII) activity and gradually terminate subsequent H_2_ production. To decrease intrinsic hydrogenase oxygen sensitivities, scientists have also tried to construct mutants with improved oxygen tolerances [64] but limited progress has been made. So far, large-scale application of photosynthetic H_2_ production is still not realistic.

**Figure 6.**
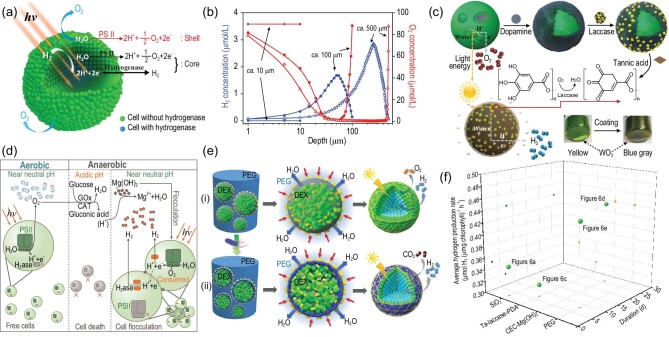
Microalgae–material hybrid for photosynthetic H_2_ production. (a) Spatial–functional differentiation in the silicification-induced *Chlorella* aggregate for photosynthetic H_2_ production under aerobic conditions. Reproduced with permission from [22]. Copyright 2015 John Wiley & Sons, Inc. (b) H_2_ (blue) and O_2_ (red) microprofiles of *Chlorella* aggregates with different sizes. Reproduced with permission from [22]. Copyright 2015 John Wiley & Sons, Inc. (c) Schematic illustration of the construction of the laccase–PDA-coated *Chlorella* cells for H_2_ production under aerobic conditions. Reproduced with permission from [67]. Copyright 2019 John Wiley & Sons, Inc. (d) Schematic of the chemoenzymatic cascade (CEC) strategy for sustainable photosynthetic H_2_ production under anaerobic conditions with *Chlamydomonas reinhardtii.* Reproduced with permission from [68]. Copyright 2020 Royal Society of Chemistry. (e) Schematic illustration of the assembly and spatial organization of *Chlorella*/*Escherichia coli* hybrid spheroids for H_2_ production under aerobic conditions [69]. (f) Hydrogen production rate and duration of microalgae–material hybrid [22,67–69].

At present, the research on microalgae photosynthetic hydrogen production mainly focuses on the single-cell level. However, many biological processes and functions in nature involve multicellular aggregation. Aggregation can affect the solute flow and communication between cells, and thus lead to the production of biological characteristics that are different from those of a single cell [66]. It was found that silicification-induced *Chlorella* aggregates could achieve sustainable photosynthetic hydrogen production under natural aerobic conditions for 60 h [22]. The sizes of the *Chlorella* aggregate could be controlled in the range of 10–500 μm by regulating the cell density in the silicification medium and the H_2_ production rate of the 100-μm aggregates can reach ∼0.35 μmol H_2_ h^−1^(mg chlorophyll)^−1^ (Fig. 6f) [22]. The aggregate can be considered to be an artificially induced multicellular organism with differentiated cell functions (Fig. 6a) [22]. Here, H_2_ and O_2_ microsensors were used to examine the interior environment in the aggregates and the profiles indicated that H_2_ was evolved by the inner cells rather than the surface cells (Fig. 6b) [22]. The surface cells retained the native photosynthetic functions and acted as autogenous shells to prevent the penetration of light into the aggregate core, while the inner cells consumed diffused O_2_ and photosynthesis-evolved O_2_ through respiration, thus creating a hypoxic domain in the core. The anaerobic condition was mainly a result of the dynamic balance between photosynthetic oxygen evolution and cellular respiration. The PSII activity and hydrogenase activity could be simultaneously maintained in this domain, which guaranteed sustainable photosynthetic H_2_ production. Our study provides a novel approach to suppressing O_2_ production for sustained photobiological H_2_ production. More generally, a similar cell–material hybrid may be used in other microorganisms for inducing functional transformation.

Blocking O_2_ production from PSII will restrict photosynthetic electron sources and ultimately limit photosynthetic H_2_ production. Therefore, the timely removal of O_2_ without damaging PSII may be a more promising strategy for inducing photosynthetic H_2_ production. Recently, enzyme-based oxygen-consuming reactions have been applied in inducing photosynthetic H_2_ production through MMH. Huang and co-workers proposed an O_2_ timely removal strategy by creating a laccase-modulated oxygen-consuming layer around *Chlorella* cells for photosynthetic H_2_ production (Fig. 6c) [67]. The laccase-based oxygen-consuming layer was sandwiched between the polydopamine (PDA) layer and the tannic acid (TA) layer, which created an anaerobic balance between photosynthetic O_2_ evolution and respiratory O_2_ consumption; then the hydrogenase was activated, resulting in the switching from photosynthetic O_2_ evolution towards H_2_ production. In the presence of TA, the laccase-catalysed oxygen-consuming reaction enabled the formation of an anaerobic microenvironment around the individual *Chlorella* cell to trigger H_2_ production under bulk aerobic conditions. By controlling the amount of TA in the system, the H_2_ production could be turned on and off on demand. Significantly, the laccase-PDA-coated *Chlorella* showed little decrease on the PSII activity and continuously produced H_2_ at an average rate of 0.32 μmol H_2_ h^−1^ (mg chlorophyll)^−1^ for 7 d (Fig. 6f) [67].

In addition to laccase, glucose oxidase could also used for timely removal of O_2_ but the oxygen-consuming reaction catalysed by glucose oxidase could cause an acidic environment, which leads to hydrogenase inactivation and even cell inactivation. To solve this problem, Fan and co-workers constructed a chemoenzymatic cascade (CEC) system for creating an anaerobic and constant near-neutral environment for sustainable hydrogen production of *Chlamydomonas reinhardtii* flocculation (Fig. 6d) [68]. The *C. reinhardtii* could stably produce hydrogen in the CEC system for 26 days, with an average rate of 0.44 μmol H_2_ h^−1^(mg chlorophyll)^−1^ (Fig. 6f) [68]. In the CEC system, four components were employed in creating the appropriate anaerobic environment: glucose oxidase (GOx), catalase (CAT), glucose and Mg(OH)_2_. The photosynthetic O_2_ can react with glucose under the catalysis of GOx/CAT. The intermediate gluconic acid may cause a decrease in pH and suppress the activity of *C. reinhardtii*. To maintain the near-neutral environment, the Mg(OH)_2_ played an important role in neutralizing the gluconic acid. In addition, Mg(OH)_2_ was also used as a flocculant for *C. reinhardtii* aggregation, which was helpful for creating an anaerobic environment. In this study, CEC played a significant role in quickly removing O_2_, while cell flocculation made a contribution to suppressing O_2_ production. Due to the synergistic effects of CEC and flocculation, an anaerobic environment for stable photobiological H_2_ production of ≤26 days was achieved (Fig. 6d and f) [68]. The CEC system helped to maintain the appropriate anaerobic environment without damaging microalgae activity or reducing chlorophyll content, which is key to sustainable photosynthetic H_2_ production. This study opens a new avenue to sustainable photosynthetic hydrogen production through rapidly removing O_2_ coupled with suppression of O_2_ production.

Enzyme-based oxygen-consuming reactions not only require harsh reaction conditions, but also have high economic costs. Therefore, the enzyme-based oxygen-consuming reaction may not be suitable for commercial application in inducing photobiological hydrogen production. Apart from oxidases, some bacteria can also consume oxygen through their respiration. Based on bacteria respiration, dextran-in-PEG emulsion micro-droplets-based algal/bacterial cell communities were developed for photosynthetic hydrogen production in daylight under air (Fig. 6e) [69]. The localized hypoxia induced hydrogen production in the core of the algal/bacterial microdroplet and demonstrated that photosynthetic hydrogen production can be promoted by enclosing the algal cells within a shell of aerobic bacterial cells. In this way, hypoxic photosynthesis and aerobic respiration were coupled synergistically to produce hydrogen from the spatially organized algal/bacterial community. The *Chlorella*/*Escherichia coli* hybrid spheroids continuously produced H_2_ at an average rate of 0.44 μmol H_2_ h^−1^ (mg chlorophyll)^−1^ for 8 days (Fig. 6f) [69]. The similarity between the above work is combining microalgae with oxygen-consuming living materials to create an anaerobic environment, which offers a new material strategy for inducing microalgae photosynthetic H_2_ production.

MMHs have been an emerging strategy for inducing microalgae photosynthetic H_2_ production under natural aerobic conditions, although current methods focus on reducing oxygen content in the microenvironment. With the progress of material technology, hydrogen production rates and the duration of MMHs have been both increasing (Fig. 6f). We firmly believe that microalgal aggregates have enormous potential in overcoming the bottleneck of sustainable photosynthetic hydrogen production in the future. Although the present approaches are far from practical application, MMH technology offers endless possibilities for improving photosynthetic H_2_ production either from the perspective of microalgae or materials, or from the perspective of microalgae–material interactions.

### Augmenting bioelectrochemical energy conversion

From the perspective of energy conversion, the essence of photosynthesis is the conversion of light energy into chemical energy through electrical energy. In photosynthesis, PSII harvests light energy and splits water to generate electrons, then the photosynthetic electrons transfer into high-energy compounds through the photosynthetic electron transport chain (PETC). Therefore, the process of photosynthesis is accompanied by biological currents. However, the bioelectricity only exists inside cyanobacterial cells or chloroplasts. The photosynthetic electrons must be transferred across the cytoplasm to reach the cytoplasmic membrane and exported to external electrodes if the intracellular bioelectricity is to be harnessed. PSII-based electrodes have been used for achieving photocurrents, but PSII is very sensitive to light and can self-repair every ∼15 min *in vivo*. In contrast, biophotovoltaic systems (BPVs), which are based on microalgae–electrode hybrids [70], offer bioelectrochemical energy-conversion technology with enhanced stability and longevity.

**Figure 7.**
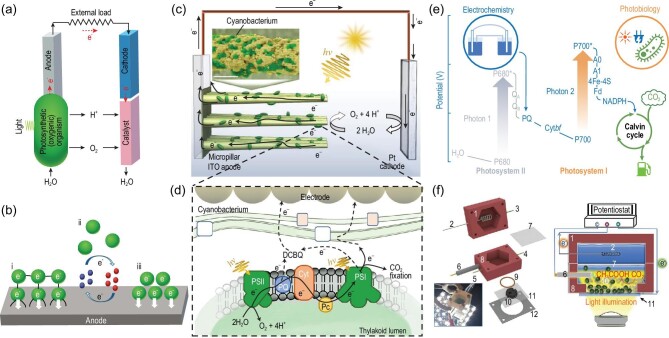
Microalgae–material hybrid for bioelectrochemical energy conversion. (a) Microalgae-based biophotovoltaic systems (BPVs). (b) Mechanisms of microalgae–material electron transfer in bioelectrochemical systems: (i) direct contact by pili between cells and electrode, (ii) indirect contact and electron transfer by endogenous electron mediators or artificial electron mediators (green sphere represents microalgal cell, blue and red spheres represent electron shuttles), (iii) direct contact and electron transfer by surface redox proteins. (c) Schematic illustration of a bioelectrochemical cell with hierarchical pillar array electrodes using cyanobacterial biofilms as photocatalysts. Inset is the scanning electron micrographs (SEM) image of a micropillar with a cyanobacteria-adsorbed electrode printed from ITO nanoparticles. Scale bar, 10 μm. Reproduced with permission from [75]. Copyright 2022 Nature Publishing Group. (d) Schematic illustration of the cyanobacterium–anode interface. Reproduced with permission from [75]. Copyright 2022 Nature Publishing Group. (e) Electron transport mechanism of microalgae-based electrophototrophic system for CO_2_ valorization with external supply of light and electricity. Reproduced with permission from [85]. Copyright 2021 Royal Society of Chemistry. (f) Schematic illustration and photograph (inset of left panel) of the cyanobacteria-based electrochemical device for CO_2_-to-acetate conversion. Component 1: polytetrafluoroethylene (PTFE) anodic part; Component 2: platinum counter electrode; Components 3–5: medium inlet/outlet; Component 6: Ag/AgCl reference electrode; Component 7: Nafion membrane; Component 8: PTFE cathodic part; Component 9: seal O-ring; Component 10: carbon felt; Component 11: fluorine-doped tin oxide (FTO) glass; Component 12: working electrode clamp. Right panel shows the electrons delivery process of cyanobacteria-based CO_2_-to-acetate electrochemical conversion. Reproduced with permission from [85]. Copyright 2021 Royal Society of Chemistry.

The BPV device is composed of an anode and a cathode, like an ordinary battery. Bioelectrochemical energy conversion in BPVs includes three basic steps, which are photosynthetic electron generation, transport of electron to the anode and cathodic reduction of oxygen (Fig. 7a) [70]. In the anodic side, the oxidation reaction occurs via a photosynthetic process: photosynthetic electrons transfer from the thylakoid membrane to the cytoplasmic membrane, then transfer from the cytoplasmic membrane to the anode electrode [70]. In the cathode side, the reduction reaction is performed by using photosynthetic oxygen as an electron acceptor [70]. The overall activity of BPVs depends on the redox reactions, so the transfer of electrons from microalgae to the anode in BPVs is the key to bioelectrochemical energy conversion. The main principle is based on the electron transfer from anode to cathode through an external circuit, whereas protons diffuse through the cytoplasmic membrane to reduce oxygen at the cathode (Fig. 7a) [70]. As cyanobacteria are prokaryotes, the photosynthetic electrons cross fewer barriers to reach the cell membrane surface in a cyanobacteria cell than in an eukaryotic algae cell, so cyanobacteria are more commonly used in BPV research.

The electron transportation in BPVs is an extracellular process in which electrons are usually transferred to the anode directly via cytochromes located in the cell membranes (Fig. 7b) [70]. There are three electron-transfer mechanisms in BPVs: (i) direct electron transfer through pili, (ii) indirect electron transfer through electron shuttling, (iii) direct contact and electron transfer through surface redox proteins (Fig. 7b). Some cyanobacteria species have pili that can directly transfer electrons to the external anode [71]. In BPVs, pili can act as ‘nanowires’ to transfer electrons between the cell membrane and the anode surface (Fig. 7b) [71]. For example, the *Synechocystis* sp. PCC 6803 cells with pili are better wired to the electrode [72–75]. Pili mainly control cell communication and phototaxis in cyanobacteria and also can be used as conductive wires [71]. Electron shuttling is another electron-transfer mechanism that involves cyclic diffusion of the soluble mediator into the microalgal cell after oxidizing at the anode (Fig. 7b) [76]. In addition, direct electron transfer on a cell–material interface can also occur through direct contact between the cell and the anode with the help of redox proteins on the cell surface (typically c-type cytochromes) [77].

The bottleneck in BPVs is electron-transfer efficiency from the photosystems to the anode across the cytoplasmic membrane and periplasmic membrane [70]. Currently, there are three main approaches to improving the electron-transfer efficiency: (i) modification of the photoanode structure, (ii) regulation of the photoanode–microalgae integration and (iii) modification of the microalgae [70,78]. Photoanodes have undergone evolution from flat structures to nanoporous structures and then to microporous structures. It was demonstrated that porous structures offered a large electroactive surface area with aided biocatalyst penetration [79,80], although porous structures were not conducive to mutual contact between microalgae and electrodes. The state-of-the-art photoelectrochemical electrode architecture is an inverse opal (IO) porous structure made from indium tin oxide (ITO) [79,81] because ITO material can exhibit appropriate inertness, excellent conductivity, light scatterability and biocompatibility [79]. Recently, an aerosol jet printing method for constructing hierarchical pillar array electrode structures using ITO nanoparticles was developed for semi-artificial photosynthesis in cyanobacteria-based BPVs (Fig. 7c and d) [75]. The micropillar array electrodes with microbranches were favorable for cyanobacteria loading, light utilization and electron flux output, so they almost doubled the photocurrent of the state-of-the-art porous structures with the same height [75]. When the heights of the micropillars increased to 600 μm, the mediated photocurrent outputs could reach 245 μA cm^–2^ (the closest to theoretical predictions) with an external quantum efficiency of ≤29% [75]. Apart from electrode structures, biofilm structures also affect microalgae–electrode interactions. For example, digitally printed cyanobacteria thin films were also developed for generating a sustained electrical current that was observed to be stable during a 10 h light/14 h dark cycle for 4 days [74]. Further, synthetic biology approaches can not only modify microalgae surface, but also promote photosynthetic electron generation, which enhances bioelectricity from the source [78]. In order to investigate cell–electrode interaction, a host of techniques based on the simpler platforms (electrochemical impedance spectroscopy, confocal fluorescence microscopy, surface-enhanced Raman/infrared spectroscopy, transient absorption spectroscopy and nanoelectrodes) have been established that can provide a wealth of underlying information for BPV systems [82,83].

Although BPV is a promising semi-artificial system for bioelectricity and fuel generation, it is not practical to generate electricity on a large scale in the short term due to its high cost and low efficiency. However, it may be easier for microalgae–electrode hybrids to be applied by introducing external electrons into the PETC for selective synthesis of high-energy compounds. Recently, electrochemically driven photosynthetic electron transport in cyanobacteria lacking PSII has been demonstrated [84] and an integrated electron-transfer strategy that converts external electrical energy into biochemical energy has also been developed [85]. The integrated photoelectrochemical architecture shuttles electrons directly to the PETC in living cyanobacteria and drives CO_2_ fixation with high energy efficiency (Fig. 7e) [85]. The cathode interfaces with PETC-modified cyanobacterial cells that lack photosystem II (PSII) activity and cannot perform photosynthesis alone [85]. Illumination of the cathode transfers electrons from an external circuit to the intracellular PETC through PS I, ultimately driving the conversion of CO_2_ into acetate in cyanobacteria (Fig. 7f) [85]. The energy-conversion efficiency for acetate production reached ∼9% under exogenous electron supply and programmed intermittent LED illumination (400–700 nm) when the numbers of photons and electrons received by the cyanobacteria were both taken into account [85]. This approach is applicable for CO_2_ reduction by using engineered cyanobacteria and has the potential to produce hydrocarbon fuels from the Sun and CO_2_.

The microalgae–electrode hybrid offers the prospect of financially feasible light energy capture-and-conversion technology, along with the added benefits of carbon neutrality. In the future, BPV technology can be applied in environmental bioenergy extraction, landscape architecture power generation, micro-battery chips and so on. The development of MMH technology will improve the efficiency of biophotovoltaic power generation and photoelectrochemical biosynthesis, and provide a carbon-neutral approach for primary energy utilization and secondary energy production.

### Boosting biochemical energy conversion

**Figure 8.**
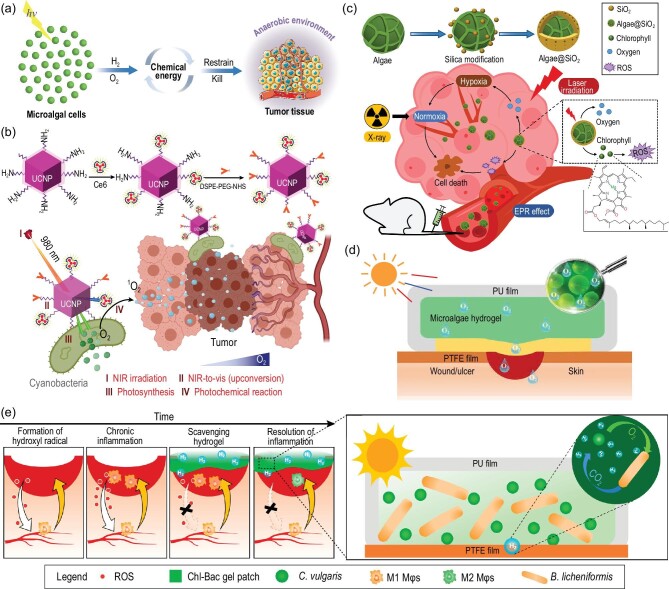
Microalgae–material hybrid for biochemical energy conversion. (a) Schematic illustration of microalgae–tumor biochemical energy conversion for biomedical therapy. (b) Schematic illustration of cyanobacteria–UCNP-Ce6 for enhanced photodynamic therapy of hypoxic tumors. Reproduced with permission from [95]. Copyright 2021 Springer Nature. (c) Schematic illustration of the *Chlorella*–SiO_2_ hybrid for cascade radio-photodynamic therapy. Reproduced with permission from [97]. Copyright 2020 American Chemical Society. (d) Schematic illustration of microalgae–hydrogel patch for chronic wound healing by light response dissolved oxygen release [25]. (e) Schematic illustration of the algae−bacteria gel patch for producing H_2_ to modulate oxidative stress in diabetic wound healing. Reproduced with permission from [24]. Copyright 2022 American Chemical Society.

From the perspective of energy metabolism, pathological cells are caused by energy deficiency, energy transmission obstacles and energy metabolic disorders [86,87]. Take tumor cells, for instance—the tumor microenvironment is not only anaerobic, but also rich in free radicals. Microalgae photosynthesis can not only evolve a large amount of oxygen to relieve tissue hypoxia, but also produce hydrogen to scavenge free radicals. Therefore, we propose that microalgae can transfer light energy to tumor cells in the form of chemical energy, which will have a therapeutic effect in tumor therapy (Fig. 8a). Due to the existence of an immune mechanism in the human body, the direct delivery of microalgae cells into tissues will cause rejection and cellular damage, so it is necessary to camouflage their cell surfaces. Inspired by the basics of the Trojan Horse, researchers consider that MMH may offer a promising strategy of engineering microalgae for alleviating immunoreactions in cross-species energy conversion.

Local hypoxia in solid tumors is considered a major cause of intolerance to many conventional cancer therapies, particularly for photodynamic therapy (PDT) and radiotherapy (RT), in which oxygen is involved in the process of cell killing [88,89]. Relieving and overcoming tumor hypoxia could substantially enhance PDT/RT efficacy. Thus, reoxygenation of hypoxic tumors has been an effective approach to overcoming hypoxia-based resistance to some conventional cancer therapies [90,91]. Although various nanocarriers have been investigated for *in situ* oxygenic production in tumors, these approaches have been limited due to the serve toxicity of the chemical compositions and immunological reactions in biological systems [92,93]. Due to effective photo-controlled oxygen evolution and good biological safety, microalgae have attracted great interest in the *in vivo* treatment of multiple diseases [94–99].

Currently, cyanobacteria and *Chlorella* are most commonly used in enhancing PDT/RT efficacy. Cyanobacteria coated with upconversion nanoparticles (UCNPs) were constructed for improving the efficiency of PDT for hypoxic tumors (Fig. 8b) [95]. In PDT, chlorin e6 (Ce6) is a widely used photosensitizer [95] but it requires an excitation wavelength of 660 nm, which is ineffective for tumor penetration. When irradiated with excitation light at 980 nm, the UCNPs on the cyanobacterial cell surface convert near-infrared (NIR) light into visible light, which can be absorbed by cyanobacteria (400–700 nm) and Ce6 (660 nm) [95]. Thereby, the Ce6 can use photosynthetic oxygen evolved by cyanobacteria to produce singlet oxygen for PDT [95]. The survival rate for the tumor-bearing mice with cyanobacteria–UCNP–Ce6 hybrid under NIR light reached 100%, which demonstrated that the cyanobcyeria–UCNP–Ce6 had an excellent antitumor effect under NIR light irradiation [95]. Due to the excellent photosynthetic oxygen-release capacity and abundant chlorophyll content, the living *Chlorella* cells were also used to produce oxygen in the tumor site to promote the radiation responses of the tumor [96–99]. The *Chlorella@*SiO_2_ hybrid was synthesized by using a one-step biomimetic silicification method that could significantly enhance the cytotoxicity and tolerance of the *Chlorella* cells, improving their living activity in the tumor area (Fig. 8c) [97]. The obtained *Chlorella*@SiO_2_ was coated by a silica shell with a thickness of 100−200 nm [97]. *In situ* oxygen generation in tumor sites by *Chlorella*@SiO_2_ photosynthesis could relieve tumor hypoxia, resulting in the enhanced efficiency of radiation therapy [97]. As a natural photosensitizer, the released chlorophyll from *Chlorella*@SiO_2_ could produce ROS to kill the tumor cells for the cascaded PDT (Fig. 8c). For *Chlorella*@SiO_2_-mediated RT combined with PDT, the tumor volume was reduced by >90% in the mice, which demonstrated the promising antitumor effect of *Chlorella* [97]. In addition, the *Chlorella* cell also can be engineered with a calcium phosphate (CaP) shell to greatly regulate the hypoxia milieu in tumor tissues [98]. The CaP shell on the surface of *Chlorella* can provide an immune shield in a hostile microenvironment, leading to oxygen generated *in situ* and ROS for alleviating the hypoxia condition of tumor tissues and killing cancer cells [98]. Although synthetic materials can provide camouflage encapsulation for microalgae, they can also cause body rejection. In order to reduce the body rejection, the red blood cell membrane (RBCM) was used to modify the surface of the *Chlorella* [99]. The RBCM-coated *Chlorella* can efficiently generate O_2_*in situ* with their natural photosynthetic system, which can alleviate the tumor hypoxia condition of tumor tissues [99]. The production of ROS from RBCM-coated *Chlorella* through laser irradiation can not only enhance the therapeutic effect of RT, but also improve the killing of cancer cells in PDT [99]. In the above studies, the MMH only provided auxiliary enhancement for tumor therapy, but could not directly treat the tumor. Besides, how the MMH gets out of the body is also a problem for clinical safety.

Apart from tumors, chronic wounds in diabetes also encounter a hypoxia microenvironment, which may result in damaged neovascularization [25]. How to relieve hypoxia in chronic wounds is a key clinical problem in diabetic disease treatment. However, traditional methods such as hyperbaric oxygen inhalation and topical gaseous oxygen therapy are limited in clinics owing to insufficient oxygen in the wound site. Therefore, microalgae-based *in situ* oxygen therapy was proposed as a new effective approach [24,25]. For instance, a patch-like wound dressing made of gel beads containing active cyanobacteria could be utilized for tissue repair in diabetic chronic wounds [25] (Fig. 8d). A skin flap of a diabetic mouse with the treatment of an alga-gel patch was completely healed in 6 days [25]. The cyanobacteria in a wound dressing can provide topical dissolved oxygen to human skin and growth factors, which can enhance wound oxygenation, fibroblast proliferation and angiogenesis to improve the healing effect [25]. In addition, excessive ROS produced by hyperglycemia or chronic inflammation can also limit diabetic wound healing [100]. H_2_ is a widely used antioxidant for scavenging ROS [100,101], so microalgae-based *in situ* hydrogen therapy may be a feasible approach to promoting diabetic wound healing. Recently, Wu and co-workers developed a H_2_-producing hydrogel made of symbiotic *Chlorella*–*Bacillus*, which can selectively reduce toxic •OH and ONOO− species and relieve inflammation, thereby promoting the healing of chronic wounds by almost 50% at Day 3 [24] (Fig. 8e). Moreover, a bioactive cyanobacteria hydrogel made by loading berberine (BBR, a quorum sensing inhibitor and antibacterial agent) can also promote infected-wound healing in diabetic mice through interrupting the quorum sensing of bacteria, relieving hypoxia and destroying biofilms [102]. Considering that the living microalgal gel could be used in other treatments, such as cytokine therapy and non-invasive stem cell therapy, the microalgal wound dressing may also be used in translational and regenerative medicine, such as tissue and cell regeneration.

Generally, both tumor and inflammatory cells are energy-intensive, while microalgal cells are energy-producing. The MMH can convert high-quality light energy into low-quality chemical energy through photosynthesis, and the chemical energy can spontaneously convert into bioenergy in tumors and inflammatory tissue. Therefore, the MMH for biochemical energy conversion offers a new strategy for biomedical therapy that is based on energy conversion. It should also be noted that current research mostly involves the improvement of existing therapies through MMHs, but they are not yet able to fundamentally transform diseased tissue. Recently, Tang and co-workers developed a therapeutic strategy for degenerative diseases based on nanothylakoid units (NTUs), which can enter chondrocytes and enhance cell anabolism by providing ATP and NADPH independently [103]. To enable cross-species, a specific mature cell membrane (the chondrocyte membrane (CM)) was used for camouflage encapsulation, which can overcome the pre-existing immune mechanism in the human body. Moreover, the CM-NTUs can protect against pathological progression of osteoarthritis by systemically correcting energy imbalance and restoring cellular metabolism. What is distinctive in the study is that the therapeutic strategy for degenerative diseases is based on the natural photosynthetic system that can enter the disease cell as a transplanted organelle, while the other studies are mainly based on tissue microenvironment regulation by microalgae. This study further provides an enhanced understanding of the cross-species photosynthetic energy conversion for the treatment of disease, which may boost the application of MMHs in biotherapy. However, the biosafety and biocompatibility of microalgae in human bodies should be solved before the ultimate clinical application of microalgae-based *in situ* therapy.

## CHEMICAL MECHANISM OF MICROALGAE–MATERIAL INTERACTION

**Figure 9.**
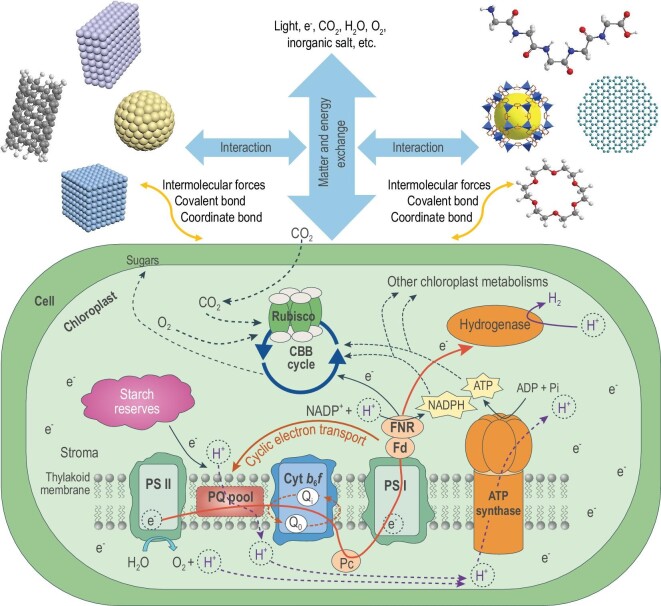
Schematic illustration of chemical mechanisms for microalgae–material interaction. Materials combine with microalgae through intermolecular forces, covalent bonds or coordinate bonds, which interfere with the exchange of matter and energy between the microalgae and the extracellular environment. The microalgae–material interface affects the photosynthetic electron transport chain through three principal pathways: (i) material hinders light energy from entering the photosystems to produce photosynthetic electrons, (ii) extracellular electrons enter the cell and are involved in photosynthetic electron transport, (iii) extracts electrons from the inner surface of the cell.

There are two levels of meaning in microalgae–material interaction: one is the material-induced construction of MMHs and the other is the material-endowed improvement of microalgal function. From a perspective of the construction of MMHs, the chemical mechanism is microalgae combined with material through intermolecular forces, covalent bonds or coordinated bonds to form the biotic–abiotic interface, and the materials affect microalgae functions by interfering with matter and energy transfer between the microalgae and the extracellular environment (Fig. 9). Inorganic materials combine with microalgal cells mainly through coordination bonds and intermolecular forces, while organic materials combine with microalgal cells mainly through covalent bonds and intermolecular forces. In terms of interaction strength, a covalent bond is stronger than coordination, stronger than intermolecular forces. However, covalent binding is not the most common method, mainly due to its effect on cell activity. Coordination bond binding can not only provide stable cell–material contact, but also keep good cell activity. Therefore, *in situ* formation of inorganic materials on the cell surface under mild conditions is a feasible and common approach to constructing MMHs.

Apart from MMH construction, microalgae–material interaction in the hybrid structure is the most critical problem. Based on the above studies, we propose two mechanisms of material-endowed improvement of microalgal function. One is microalgae–material electron transfer and the other is material-induced cell microenvironment transformation. In the mechanism of microalgae–material electron transfer, the essence of the microalgae–material interaction is that microalgae–material interface directly is involved in the PETC (Fig. 9) whereas in the mechanism of material-induced cell microenvironment transformation, the essence of the microalgae–material interaction is that the microalgae–material interface is indirectly involved in the PETC (Fig. 9). In addition, the microalgae–material interaction may also affect the respiratory electron transport chain. The microalgae–material interface not only affects cell communication with the extracellular environment, but also has a significant effect on the function of MMHs. The interface should allow efficient signal transduction (e.g. optical and electrical signals) for bidirectional cell–material communication. Therefore, it is important to develop materials with unique physicochemical properties and biological compatibility. Take BPV devices for example. Charge-transfer rates and energy-conversion efficiency have been improved by manipulating the surface chemistry of the semiconductor. However, materials are not limited to binding to the cell surface; they may also enter the microalgae cell to become artificial organelles in the future.

Investigation and understanding of the chemical mechanism of the microalgal–material interaction are the key for this research field to move from the kingdom of necessity to the kingdom of freedom. The property and function of the material must be coupled with the property and function of microalgal cells, otherwise the cell–material hybridization has no practical significance and may not be successfully achieved.

## CONCLUSIONS AND OUTLOOK

The MMH has been studied for >10 years, aiming to preserve biological function in synthetic systems for diverse applications in energy, the environment and medicine. MMHs have made considerable achievements including CO_2_ fixation, H_2_ production, bioelectrochemical energy conversion and biomedical therapy (Table 1). These achievements highlight microalgal functionalization with synthetic materials, which shows the important roles of the materials in biological evolution, providing innovative views about materials in biology. In a sense, MMHs can be considered as an artificial organism evolution strategy by using a material technique. Although great progress has been made in this emerging research field, the ideal integration of microalgae and material remains in exploration.

**Table 1.** Microalgae-material hybrid for functional improvement.

Strain	Material	Hybrid method	Function	Refs
*Synechocystis* sp. strain PCC 7002	SiO_2_	Cell immobilization	Photosynthetic CO_2_ fixation	[60]
*Synechocystis* sp. strain PCC 6803	SiO_2_	Unicellular shellization	Photoinhibition alleviation	[39]
*Chlorella vulgaris*	SiO_2_–TiO_2_	Unicellular shellization	Thermoprotection	[40]
*Synechocystis* sp. strain PCC 7002	Colloidal SiO_2_	Unicellular shellization	Size-dependent permeability	[41]
*Chlorella pyrenoidosa*	CeO_2_	Unicellular shellization	UV protection	[42]
*Chlorella pyrenoidosa*	SiO_2_	Cell aggregation	Photosynthetic H_2_ production	[22]
*Chlorella pyrenoidosa*	Tannic acid, laccase, polydopamine	Unicellular shellization	Photosynthetic H_2_ production	[67]
*Chlamydomonas reinhardtii*	Glucose oxidase, catalase, Mg(OH)_2_	Cell aggregation	Photosynthetic H_2_ production	[68]
*Chlorella pyrenoidosa* /*Escherichia coli*	PEG	Cell aggregation	Photosynthetic H_2_ production	[69]
*Chlorella vulgaris*	ITO-PET	Cell immobilization	Photocurrent production	[23]
*Dunaliella tertiolecta*	ITO-PET	Cell immobilization	Photocurrent production	[23]
*Synechocystis* sp. strain PCC 6803	ITO-PET	Cell immobilization	Photocurrent production	[23]
*Synechococcus* sp. WH 5701	ITO-PET	Cell immobilization	Photocurrent production	[23]
*Nostoc punctiforme*	Porous ITO	Cell immobilization	Photocurrent generation	[80]
*Synechocystis* sp. strain PCC 6803	Porous ITO	Cell immobilization	Photocurrent generation	[80]
Synechocystis sp. strain PCC 6803	Graphite	Cell immobilization	Photocurrent generation	[73]
*Synechocystis* sp. strain PCC 6803	Carbon nanotubes	Cell immobilization	Photocurrent generation	[74]
*Synechocystis* sp. strain PCC 6803	Pillar array IO-ITO	Cell immobilization	Photocurrent generation	[75]
*Synechocystis* sp. PCC 6803 mutant strain	Carbon felt and FTO	Cell immobilization	CO_2_-to-fuels conversion	[85]
*Synechococcus elongatus* PCC 7942	Upconversion nanoparticles (NaYF4:Yb, Er)	Unicellular shellization	Photodynamic therapy enhancement	[95]
*Chlorella vulgaris*	SiO_2_	Unicellular shellization	Radio-photodynamic therapy enhancement	[97]
*Chlorella vulgaris*	CaP	Unicellular shellization	Radio-Photodynamic therapy enhancement	[98]
*Chlorella vulgaris*	Red blood cell membrane	Unicellular coating	Radio-photodynamic therapy enhancement	[99]
*Spirulina platensis*	Sodium alginate hydrogel with carboxymethyl chitosan	Cell immobilization	Quorum sensing inhibition and infected wound healing promotion	[102]

Since microalgae and materials are two key components in constructing MMHs, the functional modification should be based on the inherent properties of microalgae. Until now, cyanobacteria (*Synechocystis* sp. strain PCC 6803, *Synechocystis sp.* strain PCC 7002 and *Synechococcus elongatus* PCC 7942) and green algae (*Chlorella pyrenoidosa, Chlorella vulgaris* and *Chlamydomonas reinhardtii*) have been the most used in the construction of MMHs and are two representative categories in the microalgae family. The cyanobacteria–material hybrid has been applied to CO_2_ fixation, bioelectrochemical energy conversion and biomedical therapy, while the green algae–material hybrid has been applied to hydrogen production and biomedical therapy (Table 1). However, there are many other kinds of microalgae to be studied for material modification. Different cells not only have different inherent properties, but also have different response mechanisms to materials, which may induce different functions of the hybrid. Optimizing the inherent properties of microalgae is also important for the hybrid to achieve better performance. For example, synthetic biology has been playing an increasingly important role in bioelectrochemical energy conversion [78]. While synthetic biology is mainly used to improve the *in vivo* function of cells [83], the tools of synthetic biology can in principle enhance the ability of microalgal cells to interact with electrodes. Future opportunities also lie in integrating synthetic materials with engineered microalgae to open up new pathways in energy conversion.

Apart from the inherent properties of microalgae, a suitable material strategy is the most critical factor to affect the function of MMHs. Here, the material strategy includes material selection and the construction method. Currently, silica is one of the most suitable materials for microalgal modification due to good biocompatibility and light transmittance, and bioinspired cell silicification is an emerging approach to induce the combination of microalgae and silica. Until now, cellular silicification has been demonstrated not only to protect cell viability against environmental stresses, such as heat, high light and UV-C irradiation, but also to induce photosynthetic H_2_ production [21,36,104]. Apart from silica, hydroxyapatite, Fe_3_O_4_, rare earth materials and hydrogel can also be used for microalgae modification. *In situ* biomimetic material formation on microalgae cell surfaces is a feasible approach to constructing MMHs, but there remain many open questions and challenges. First, the centrifugal operation and combination process inevitably cause cell damage, hence biocompatibility should continue to be improved. Second, a sophisticated structure of the materials on the cell has not been achieved. Third, most modifications by materials can prevent cell replication, but the improvement usually disappears as the cells divide, which means a lack of recyclability. Fourth, the lifespan of MMHs is limited and depends on the intrinsic lifetime of the microalgae and the protective capability of the hybrid. In addition, the construction method of the cell–material is another aspect of the materials strategy that has a great influence on the microalgal function. According to the current material technology, there are three construction methods for MMHs, including cell immobilization, unicellular shellization and multicellular aggregation. Among the three construction methods, cell immobilization of microalgae was mainly applied in CO_2_ fixation, bioelectrochemical energy conversion and biomedical therapy; unicellular shellization of microalgae was mainly applied in CO_2_ fixation, hydrogen production and biomedical therapy; while multicellular aggregation of green algae was mainly applied in hydrogen production. Except for microalgae–electrode hybrids, current modification by materials is mainly based on the changes in the cellular microenvironment induced by the material–microalgae interaction, although the material cannot confer new functions to the microalgae. Therefore, a new material strategy is critical to exploring new functions and new applications. What calls for special attention is that the MMH generally follows a principle: the selection of materials and the hybridization methods should depend on the expected function. Besides biocompatibility, stability under light conditions also needs to be considered. The advancement of nanomaterial technology is an important driving force for the development of this field. If the controllable formation and combination of nanomaterials at the biological interface can be realized, it will be helpful to realize the precise transformation of biological bodies.

Looking back on the development of MMHs in the past 10 years, we can find that the initial applications were focused on photosynthetic energy conversion. At present, the main limitation of MMHs for photosynthetic energy conversion is the low energy-conversion efficiency, which makes practical application in carbon neutrality difficult. Besides, the high cost of the microalgae culture and the lack of material technology suitable for the large-scale engineering of microalgae are also important limiting factors for practical application. In the future, improving the energy-conversion efficiency is the general aim for fundamental research. Before achieving the general aim, some fundamental questions surrounding the biological functionalization should be investigated, such as the effect of synthetic materials on microalgae at the genetic level. Besides the studies in the biological field, more work is needed in developing new techniques for integrating synthetic materials with biological systems. The important consideration in constructing the hybrid is the interface between the synthetic materials and the biological systems, not only from the perspective of biocompatibility, but also from the perspective of functional compatibility. Exploring new functions of MMHs and new application scenarios of the established hybrid systems is also a future trend of the emerging area. In some special circumstances, cost is not an issue, so appropriate application scenarios will promote the practical application of MMHs. For example, cross-species energy conversion for therapy is becoming an emerging area that mainly utilizes photosynthetic energy carriers. Meanwhile, exploring new functions and applications will spur the study of the interaction mechanism between organisms and materials.

Generally, MMHs for photosynthetic energy conversion is an emerging field and the biotic–abiotic interface is the fundamental problem in the field. The investigation of microalgae–material interaction mechanisms can not only enhance the understanding of the biotic–abiotic interface, but also promote the development of the applied research of MMHs. The MMH is not only to demonstrate material-based microalgae modification, but also to achieve functional synergy between materials and microalgae. The original intention of the MMH was to expand the application of microalgae by materials, but the material-based organism improvement has also blurred the boundary between biotic and abiotic systems, which follows the significant roles of materials in biological evolution. Furthermore, research on the biotic–abiotic hybrid can combine biological and material sciences together for an inseparable integration. Once the photosynthetic energy-conversion efficiency is achieved, MMHs can greatly promote the applications of photosynthetic CO_2_ fixation and H_2_ production. Finally, there is sufficient reason to believe that application of MMHs will greatly contribute to the achievement of carbon neutrality.
